# Supplementary material for: Peritectic titanium alloys for 3D printing
Source: Nat Commun. 2018 Aug 24;9:3426. doi: 10.1038/s41467-018-05819-9 (PMC6109080; doi:10.1038/s41467-018-05819-9)
Supplement: Supplementary file 1 — Supplementary Information [file 41467_2018_5819_MOESM1_ESM.pdf]

Peritectic titanium alloys for 3D printing

Pere Barriobero-Vila[1*], Joachim Gussone[1], Andreas Stark[2], Norbert Schell[2], Jan Haubrich[1], Guillermo Requena[1,3]

[1]Institute of Materials Research, German Aerospace Center (DLR), Linder Höhe, 51147 Cologne, Germany

[2]Helmholtz-Zentrum Geesthacht, Max-Planck-Straße 1, 21502 Geesthacht, Germany

[3]Metallic Structures and Materials Systems for Aerospace Engineering, RWTH Aachen University, 52062 Aachen, Germany

*Correspondence: pere.barrioberovila@dlr.de

This supplementary information contains five supplementary figures (Supplementary Fig. 1, Supplementary Fig. 2, Supplementary Fig. 3, Supplementary Fig. 4 and Supplementary Fig. 5).

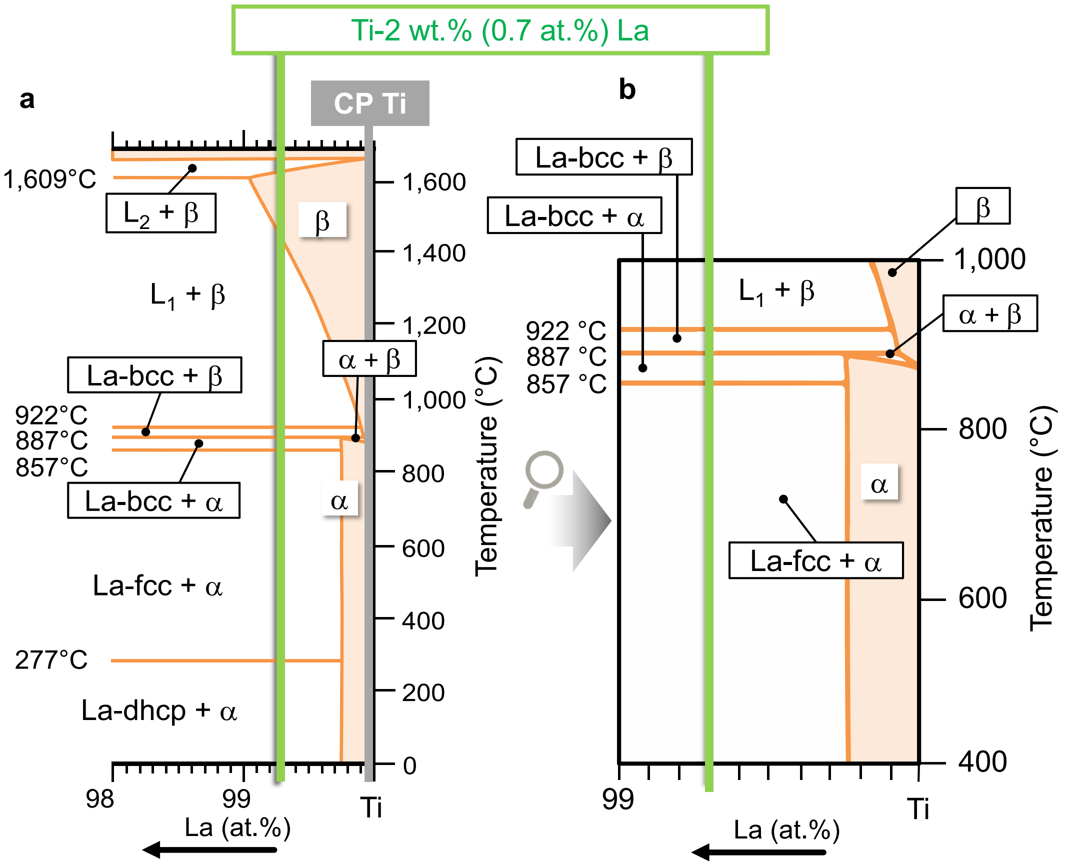

Supplementary Fig. 1 **a** Portion the Ti-La phase diagram and **b** magnified detail of the studied region adapted from[1,2], indicating the compositions used for selective laser melting.

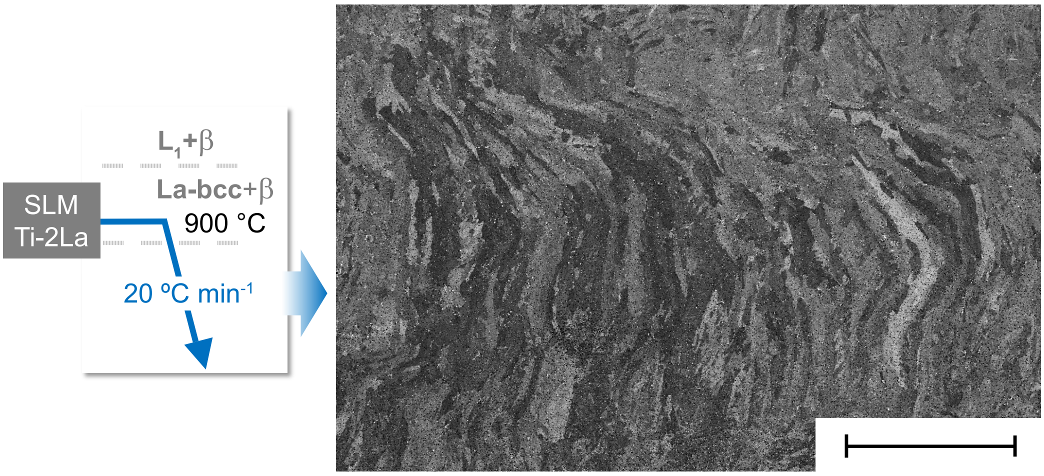

Supplementary Fig. 2 Post-thermal treatment in La-bcc+β field of the Ti-2wt.% La (Ti-2La) alloy produced by selective laser melting (SLM). Upon slow cooling of the SLM as-built Ti-2La alloy from 900°C with 20°C min^{-1} (i.e. from the La-bcc+β field down to room temperature) tortuous grain arrangements from the as-build condition remain in the microstructure, indicating negligible influence of La particles on formation of new α grains. Scale bar, 100μm.

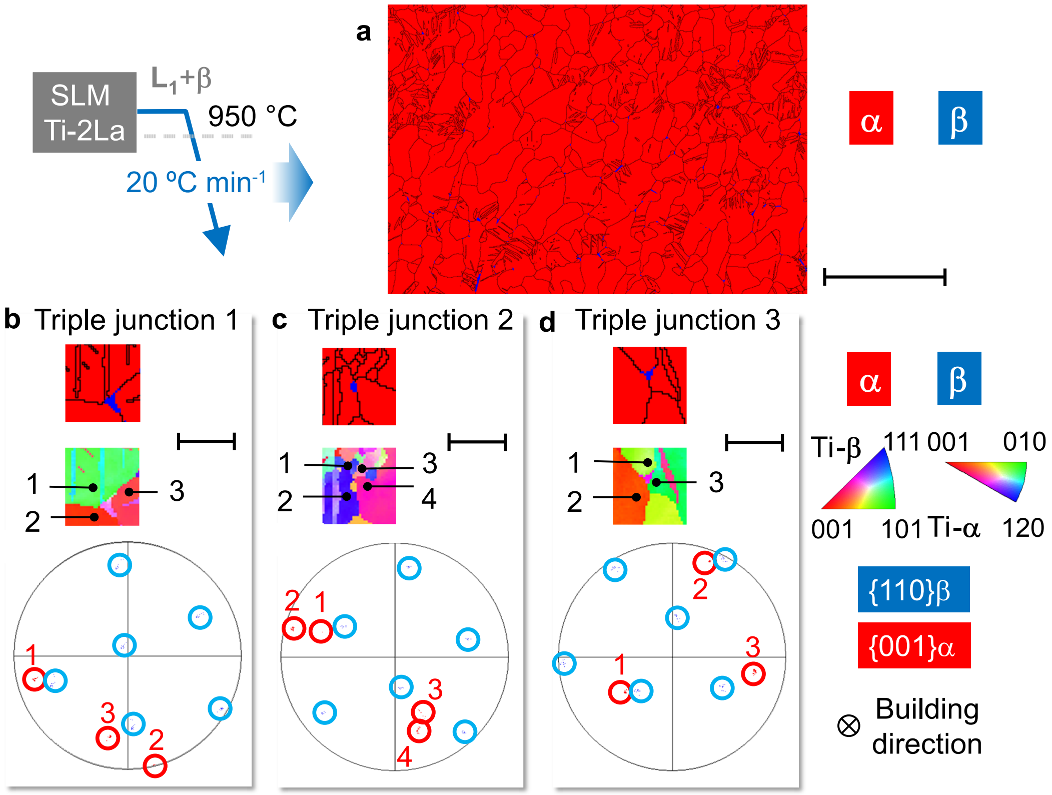

Supplementary Fig. 3 Post-thermal treatment of the SLM Ti-2La as-built condition via slow cooling with 20°C min^{-1} from 950°C passing through the peritectic line (i.e. from L_1+β field down to room temperature), provokes formation of α grains and extensive globularization. **a** EBSD phase map for α and β (in blue and red, respectively). The microstructure mainly consists in α, though retained β remains in triple junction boundaries (~1.4vol.% according to HEXRD). Scale bar, 100μm. EBSD phase maps from three sub-regions of **a** (**b** triple junction 1, **c** triple junction 2, **d** triple junction 3) are presented with its corresponding IPF maps and pole figures for {110}β as well as {001}α. Scale bars, 10μm. The α variants (encircled in red) surrounding β particles (in blue) do not present a usual Burgers OR {001}α || {110}β. This reflects the non-Burgers path of α formation taking place for the heat treated Ti-2La alloy. Black lines in EBSD phase maps of **a**, **b**, **c** and **d** indicate high angle grain boundaries (misorientation >10°).

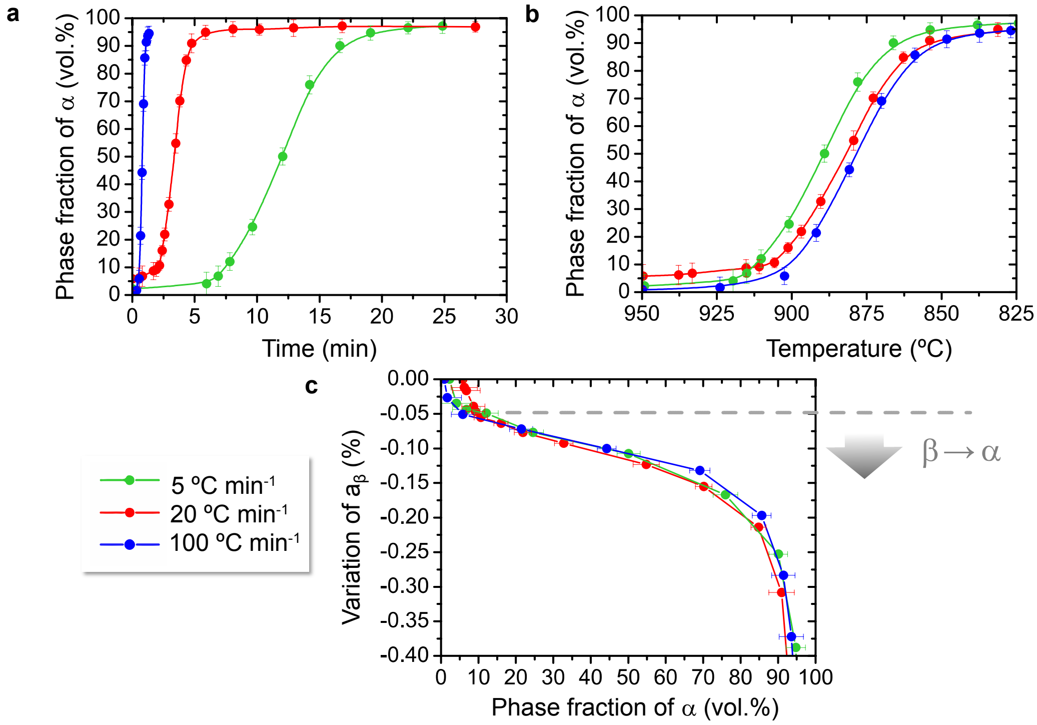

Supplementary Fig. 4 Crystallization kinetics of α in the Ti-2wt.% La alloy during continuous cooling. The evolution of volume fractions of α is presented during β → α transformation for 5, 20 and 100°C min^{-1} as a function of **a** time and **b** temperature. **c** Evolution of the variation of the lattice parameter of β, a_β, during β → α transformation as a function of the volume fraction of α for 5, 20 and 100°C min^{-1}. The error bars associated with the determination of the lattice parameters are comprised within the symbols.

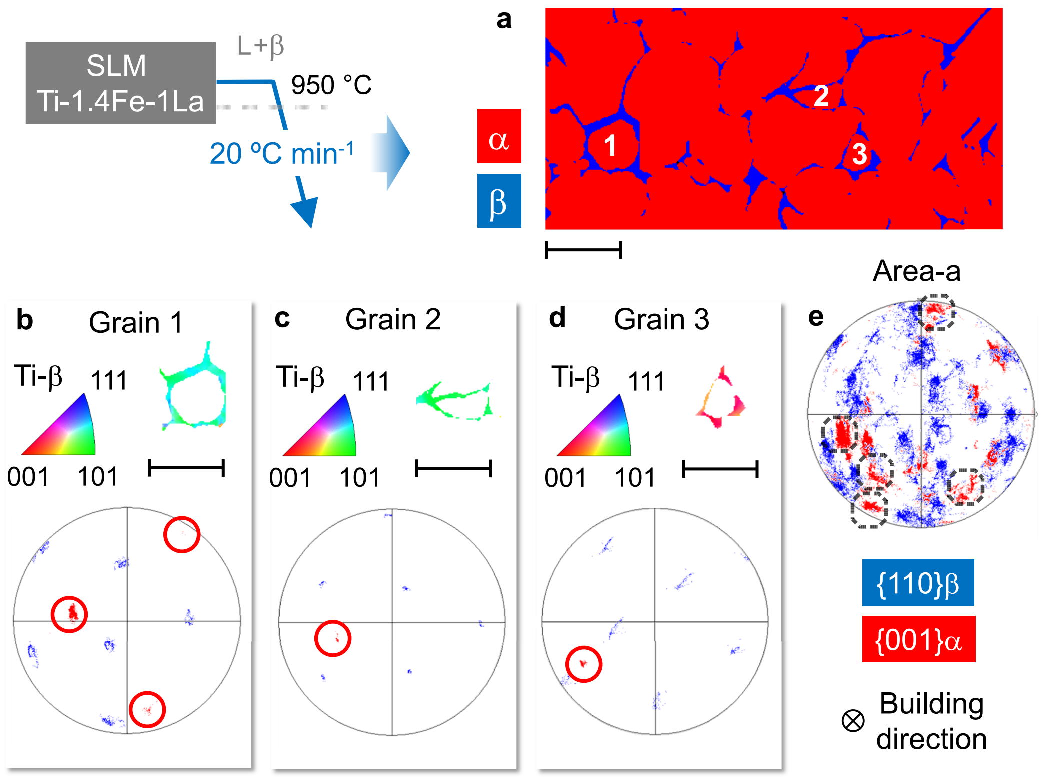

Supplementary Fig. 5 Post-thermal treatment by slow cooling the Ti-1.4Fe-1La alloy from 950°C with 20°C min^{-1} down to room temperature leads to extensive globularization of α. **a** EBSD phase map for α and β (in blue and red, respectively). Scale bar, 20μm. IPF maps of β and the corresponding pole figures for {110}β as well as {001}α obtained for the α grains pointed in **a**: **b** Grain 1, **c** Grain 2, **d** Grain 3. Scale bars, 20μm. The α variants (encircled in red) do not present a usual Burgers OR {001}α || {110}β with respect to its surrounding β matrix (in blue). This can also be observed when considering the complete area shown in **a**, where **e** encircled regions of {001}α variants (in red) do not match those of {110}β (in blue). This reflects the non-Burgers path of α formation taking place for the heat treated Ti-1.4Fe-1La alloy.

Supplementary references

1. Mattern, N. et al. Experimental and thermodynamic assessment of the La-Ti and La-Zr systems. *Calphad* **52**, 8–20 (2016).

2. Okamoto, H. Supplemental literature review of binary phase diagrams: B-Fe, Cr-Zr, Fe-Np, Fe-W, Fe-Zn, Ge-Ni, La-Sn, La-Ti, La-Zr, Li-Sn, Mn-S, and Nb-Re. *J. Phase Equilib. Diffus.* **37**, 621–634 (2016).
